# Biological Characterization of Mupirocin–KGF Hydrogel and Its Regenerative Potential in Human Fibroblast-Mediated Wound Healing

**DOI:** 10.3390/molecules30234523

**Published:** 2025-11-23

**Authors:** Sasikumar Murthy, Malarvili Selvaraja, Salah A. Alshehade, Marwan Abdelmahmoud Abdelkarim Maki, Yeun-Mun Choo, Vijayaraj Kumar Palanirajan, Vasantha Kumari Neela

**Affiliations:** 1 Department of Medical Microbiology, Faculty of Medicine and Health Sciences, Universiti Putra Malaysia, Serdang 43400, Malaysia; sasikumar.m2@gmail.com; 2 Faculty of Pharmaceutical Sciences, UCSI University, No. 1, Jalan Menara Gading, Taman Connaught, Cheras, Kuala Lumpur 56000, Selangor, Malaysia; malarvili@ucsiuniversity.edu.my (M.S.);; 3 Department of Pharmacology, Faculty of Pharmacy & Bio-Medical Sciences, MAHSA University, Petaling Jaya 42610, Selangor, Malaysia; salah@mahsa.edu.my; 4 Chemistry Department, University of Malaya, Kuala Lumpur 50603, Selangor, Malaysia; ymchoo@um.edu.my

**Keywords:** mupirocin, wound healing, fibroblast proliferation, molecular docking, MRSA, fibroblast migration

## Abstract

This study presents a novel hydrogel formulation combining mupirocin, a broad-spectrum antibiotic, with keratinocyte growth factor (KGF) to enhance wound healing through antibacterial action and tissue regeneration. Mupirocin was encapsulated in hydroxypropyl β-cyclodextrin (HP-β-CD) and stabilized with poly(amidoamine) dendrimers (PAMAM). Molecular docking studies assessed mupirocin’s binding to PAMAM and its interaction with isoleucyl-tRNA synthetase. Physicochemical properties—including zeta potential, particle size, and surface tension—were characterized, and drug release kinetics were evaluated using Franz diffusion cells. In vitro assays on human dermal fibroblasts (HS27) included proliferation, scratch wound healing, and flow cytometry to assess cellular behavior. Antibacterial efficacy was determined via the Kirby–Bauer disk diffusion method. Results showed strong binding of mupirocin to its target enzyme, enhanced by KGF. The hydrogel exhibited favorable properties: surface tension of 24.7 dyne/cm, zeta potential of −24.79 mV, and particle size of ~119 nm, indicating high stability. Franz diffusion revealed sustained drug release compared to commercial mupirocin. Cellular assays demonstrated significant fibroblast migration and proliferation, with flow cytometry confirming increased wound healing markers. The formulation showed potent antimicrobial activity, including against Methicillin-resistant Staphylococcus aureus (MRSA), highlighting its promise for infected wound treatment and advanced clinical wound care.

## 1. Introduction

An antibacterial drug called mupirocin (pseudomonic acid A) is mostly used to treat skin infections brought on by *Staphylococcus aureus*, including strains that are resistant to methicillin (MRSA) [1]. Additionally, it has efficacy against Gram-negative bacteria like *Neisseria gonorrhoeae* and *Haemophilus influenzae*, Gram-positive *cocci* like *Staphylococci* and *Streptococci* [2,3], and superficial infections brought on by dermatophytes [4]. However, its strong plasma protein binding and short systemic half-life (less than 30 min) limit its therapeutic potential by lowering its bioavailability. Its therapeutic efficacy has been limited to localized skin infections due to these pharmacokinetic limitations and increasing resistance rates (1–81%) [5,6].

Mupirocin is a strong topical antibiotic that inhibits bacterial protein synthesis by targeting isoleucyl-tRNA synthetase. Beyond its antimicrobial effect, it also stimulates keratinocyte proliferation and the secretion of key growth factors, supporting the wound healing process [7]. Because of its antibacterial action against germs linked to wounds, it has also been demonstrated to have wound-healing properties [7]. Additionally, studies have reported that conventional formulations of mupirocin have adverse effects, such as nausea, swelling, pain, skin rashes, dryness, burning, itching, redness, stinging and tenderness [8]. These limitations have reduced the duration of mupirocin therapy to up to 10 days, which does not last for longer periods of time. Therefore, novel drug delivery approaches to be used in combination with mupirocin are able to overcome the limitations of conventional formulations, mainly in terms of wound healing effects. A study reported that nano formulation using selenium nanoparticles (SeNPs) with mupirocin significantly increased the wound healing activity of a mupirocin-methicillin-resistant *S. aureus* (MMRSA)-infected rat diabetic wound model [9].

Keratinocyte growth factor (KGF) is a type of cytokine that is always used in drug formulations and has been shown to have antimicrobial and wound-healing effects by promoting epithelial proliferation, fibroblast regeneration and migration and extracellular matrix remodelling [10]. Mupirocin is a strong topical antibiotic that inhibits bacterial protein synthesis by targeting isoleucyl-tRNA synthetase. Beyond its antimicrobial effect, it also stimulates keratinocyte proliferation and the secretion of key growth factors, supporting the wound healing process [7]. These growth factors aid inflammation resolution, cell proliferation, angiogenesis, and extracellular matrix remodeling, while also helping maintain a moist environment that promotes healing and limits bacterial colonization [10,11].

While mupirocin and keratinocyte growth factor (KGF) have not yet been the subject of clinical research, their complimentary functions in tissue regeneration and infection control point to the potential of synergistic therapeutic approaches. A complex hydrogel formulation was created by encasing mupirocin in hydroxypropyl β-cyclodextrin (HP-β-CD), stabilizing it with poly (amidoamine) dendrimers (PAMAM), and combining it with KGF. It is anticipated that this combination will improve wound healing and treat bacterial infections. The current study’s objective was to assess the therapeutic potential of this mupirocin-KGF hydrogel in an in vitro fibroblast-based wound healing model, with a focus on its effects on growth factors involved in tissue repair, some of which have not been previously documented in this context.

This hydrogel is a novel combination of a broad-spectrum antibiotic and a regenerative growth factor, delivered through a multi-component system of HP-β-CD and PAMAM dendrimers. The structural complexity of this hydrogel not only improves drug stability and sustained release, but also introduces potential biointeractive properties that conventional formulations often overlook.

## 2. Results and Discussion

The integration of molecular docking, in vitro cell-based assays, and cellular analyses in this study provides a holistic understanding of the efficacy and potential of the mupirocin-KGF formulation for wound healing applications.

**Molecular Docking Analysis**

**Figure 1:**
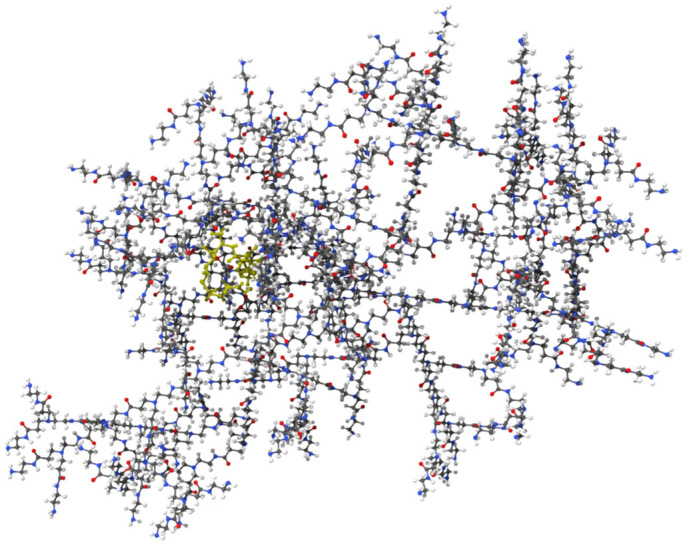
Docking results of the Mu and PAMAM dendrimers, demonstrating their encapsulation stability.

**Figure 2:**
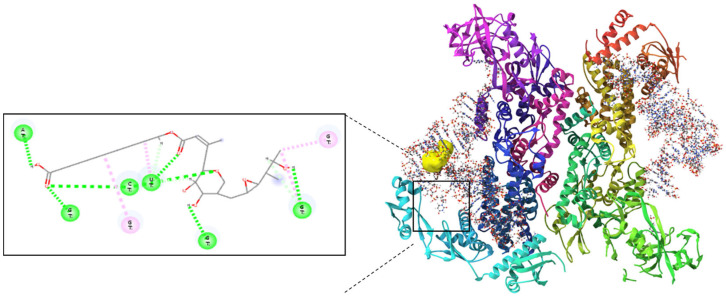
Molecular docking analysis of the mupirocin-KGF complex with isoleucyl-tRNA synthetase revealed key hydrogen bonding interactions at the active site.

**Figure 3:**
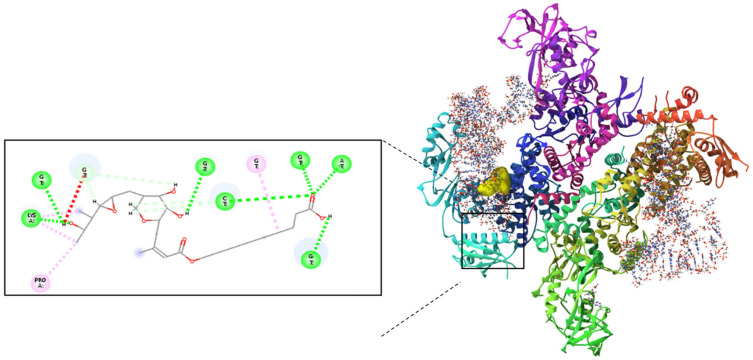
Molecular interaction of Mu with isoleucyl-tRNA synthetase, highlighting critical residues involved in the binding process.

PAMAM dendrimers are nanoscale structures with branched architectures and surface amine groups that facilitate interactions with cellular membranes and proteins. These interactions can modulate cell signalling, gene expression, and affect cellular behaviours like proliferation and migration. Research indicates that PAMAM dendrimers activate critical pathways, such as mitogen-activated protein kinase (MAPK) and extracellular signal-regulated kinase (ERK) pathways, which play significant roles in wound healing activity. Their surface characteristics also impact cellular uptake and intracellular movement, potentially influencing cytokine release and growth factor expression [12,13]. Molecular docking studies validated the complexation of Mu within the PAMAM dendrimer and assessed its ability to interact with isoleucyl-tRNA synthetase (IleRS). The binding energy of −6.42 kcal/mol for the mupirocin-KGF complex (Figure 1) confirmed successful complexation, demonstrating that Mu was stably retained within the dendrimer while maintaining its functional groups for interaction with its biological target (Figure 2). Further docking analysis revealed that the mupirocin-KGF complex exhibited a stronger binding affinity with IleRS (−7.66 kcal/mol) (Figure 2) than did pure Mu (Figure 3) (−7.58 kcal/mol) (Table 1). The improved binding affinity suggests that encapsulation did not interfere with Mu’s inhibitory effect on IleRS but instead provided a stabilizing scaffold that enhanced its molecular interactions. The key active site residues Asp 612 and Arg 826 were identified as critical binding points, where hydrogen bonding and electrostatic interactions disrupted enzyme activity. Additional residues, such as Tyr398, Gln828, and Phe026, contributed to enhanced binding via polar and hydrophobic interactions, collectively reinforcing the complex’s inhibitory potential.

Interestingly, the PAMAM dendrimer itself did not directly inhibit IleRS but instead interacted with nonactive site residues, providing peripheral stabilization to the complex. This suggests that the dendrimer acts as a passive carrier, ensuring that Mu remains in an optimal conformation for enzyme inhibition. Thus, the observed increase in binding affinity is driven primarily by Mu’s interactions with key catalytic residues, confirming that the drug remains the active inhibitory agent, whereas the structural stability and bioavailability of the PAMAM dendrimer are improved.

**Mupirocin-KGF formulation**

Hydroxypropyl β-cyclodextrin (HP-β-CD) normally has a negative zeta potential in water, which typically falls between −20 and −30 mV [14]. The quantity of mupirocin included in the hydroxypropyl β-cyclodextrin–mupirocin (HP-β-CD-Mu) complex governs the fluctuation of the zeta potential of the HP-β-CD solution. Furthermore, considering that 3G PAMAM dendrimers (third-generation polyamidoamine dendrimers) include several primary amine groups on their surface that are positively charged in aqueous solutions, they usually have a positive zeta potential in aqueous solutions, commonly ranging from +20 to +30 mV [15]. When a 3G PAMAM dendrimer complex is added to a β-CD Mu inclusive complex solution, the positive and negative charge interactions encourage the formation of microparticles in the solution. An extra 5% HPMC was added to the nanoparticulated solution to produce the naturally charged mupirocin-KGF formulation. In the nanogel formulation, glycerol serves as a solubilizing and penetrating enhancer.

**Particle size and zeta potential measurements**

Zeta potential measurement is crucial for assessing the actual electric charge present on the surface of microparticles (HP-β-CD-Mu-KGF-PAMAM-NPs) distributed in the mupirocin-KGF formulation and determining the magnitude of these charges. This measurement is important for examining the stability of the mupirocin-KGF formulation. It has a direct effect on both the toxicity of the mupirocin-KGF formulation and its initial binding to cell membranes [16]. The zeta potential, also referred to as the dispersion surface charge, is significant since it can produce strong surface charge effects that cause attractive forces between positively charged dendrimers and negatively charged polymers such as HP-β-CD and HPMV, which successfully increase the stability of the microparticles in the mupirocin-KGF formulation. Sivasankaran et al. [17] reported that greater zeta potential values signify system stability. The polarity of the zeta potential reflects the surface charge of the NPs and lower zeta potential values, indicating a propensity for NP aggregation. For example, NPs exhibiting zeta potentials exceeding +30 mV are classified as highly cationic, those falling within the range of −10 to +10 mV are considered neutral, and those with zeta potentials less than −30 mV are categorized as highly anionic.

The zeta potential of the mupirocin-KGF formulation is influenced by several variables, including pH, ionic strength, and concentration. The zeta potential is crucial for influencing the stability of the mupirocin-KGF formulation. In particular, colloids are highly stable when their zeta potential is greater than 30 mV, whereas they are quite unstable when their zeta potential is between 0 and 10 mV [18].

**Figure 4:**
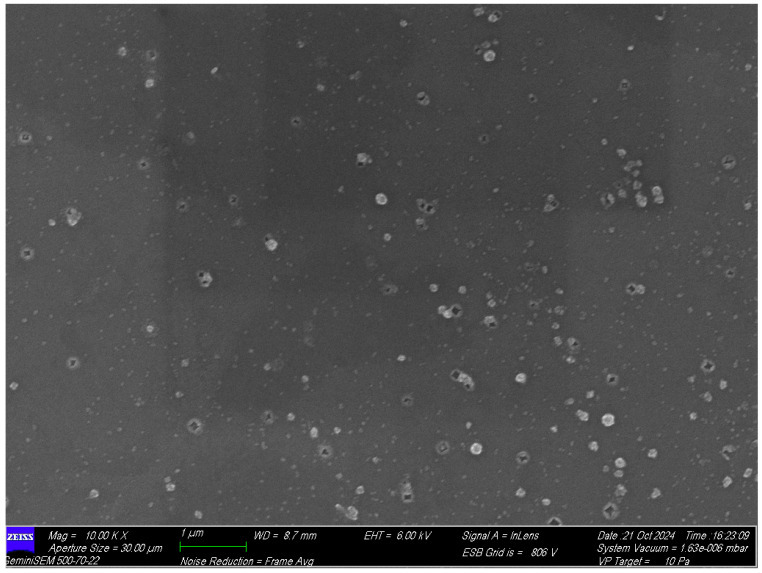
Field emission scanning electron microscopy (FESEM) images of HP-β-CD-Mu-KGF-PAMAM complex show the particles exhibit irregular polygonal shapes scattered non-uniformly across the substrate, with some clustering.

Zeta potential measurements were carried out to identify the nature of the charge present on the surface of the HP-β-CD-Mu-KGF-PAMAM-NPs present in the mupirocin-KGF formulation. Notably, the zeta potential and particle size distribution of the NPs present in the mupirocin-KGF formulation was −24.79 mV and 119.2 ± 3.43 nm (as shown in Table 2 and Figures S1 and S2). The presence of this negative surface charge indicates the exceptional stability of the mupirocin-KGF formulation. This led to their excellent dispersion in aqueous solutions. Field emission scanning electron microscopical studies revealed that the HP-β-CD-Mu-KGF-PAMAM-NPs exhibit irregular polygonal shapes scattered non-uniformly across the substrate, with some clustering shown in the Figure 4.

**The surface tension of the mupirocin-KGF hydrogel formulation**

The surface tension of the mupirocin-KGF hydrogel formulation was 24.7 dyne/cm (Table 3). Compared with water, the mupirocin-KGF hydrogel formulation has a lower surface tension. Better contact and moisture retention are essential for efficient wound healing, and this decreased surface tension makes it easier for hydrogels to spread over wound surfaces [19]. Hydrogel surface tension is crucial for wound healing and affects hydrogel adhesion and cell interactions. Ideal surface tension reduces irritation and negative responses, promoting cell migration and proliferation [20].

**In Vitro membrane permeation studies using a Franz diffusion cell**

**Figure 5:**
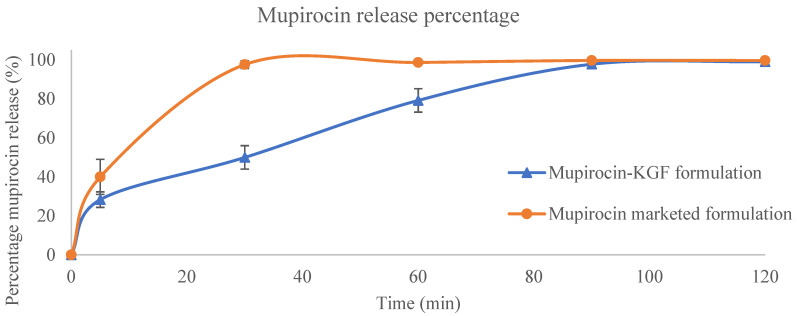
Membrane permeation profile of Mu from the mupirocin-KGF formulation in Franz diffusion studies. The data are expressed as the cumulative release (% of initial drug load) over 60 min.

The amount of mupirocin in the collected medium was determined via HPLC method and the retention time of mupirocin was found to be 1.79 min at 220 nm. In the HPLC study the excellent linearity was confirmed by establishing a linear calibration curve throughout the mupirocin concentration range of 25–250 μg/mL, which produced a correlation value of 0.9882 with a slope of 8167 and an intercept of 2,190,606.01. Using standard formulas obtained from the calibration data, detection sensitivity was evaluated, yielding a Limit of Quantification (LOQ) of 12.23 μg/mL and a Limit of Detection (LOD) of 4.041 μg/mL. Intra-day and inter-day precision tests were used to assess reproducibility; the results showed strong repeatability and acceptable intermediate precision, with %RSD values of 0.1477 and 0.1670, respectively. Compared with that of the mupirocin-KGF formulation, the total quantity of Mu that penetrated the receptor compartment of the Franz diffusion cell at various time intervals was lower and substantially different from that of the commercial formulation (*p* < 0.05) (Figure 5). This can be explained by the marketed formulation’s use of benzyl alcohol, which dissolves Mu during formulation production and facilitates a quicker influx of the drug. This finding indicated that while the commercial formulation penetrated the membrane more quickly, the presence of HP-β-CD-Mu-KGF-PAMAM-NPs in the mupirocin-KGF formulation caused the Mu to be released slowly and remain in the membrane for a longer period of time. The HP-β-CD-Mu-KGF-PAMAM-NPs distributed in the mupirocin-KGF formulation at neutral pH are quite stable because of the charge interactions between HP-β-CD (−charge), 3G PAMAM (+charge) and HPMC (−charge). Deprotonation of 3G PAMAM at pH values less than 7 occurs [21], and the interaction between 3G PAMAM and negatively charged polymers is destroyed once the mupirocin-KGF formulation diffuses through the membrane from the donor compartment of the Franz diffusion cell to the receptor compartment. This enables the full release of Mu from the mupirocin-KGF formulation at relatively low pH values.

**Cell Proliferation Analysis**

The cell proliferation response to Formula A or KGF was assessed via the CCK-8 assay over 48 h, and the concentration-dependent effects across the two different treatment groups were evaluated.

**Figure 6:**
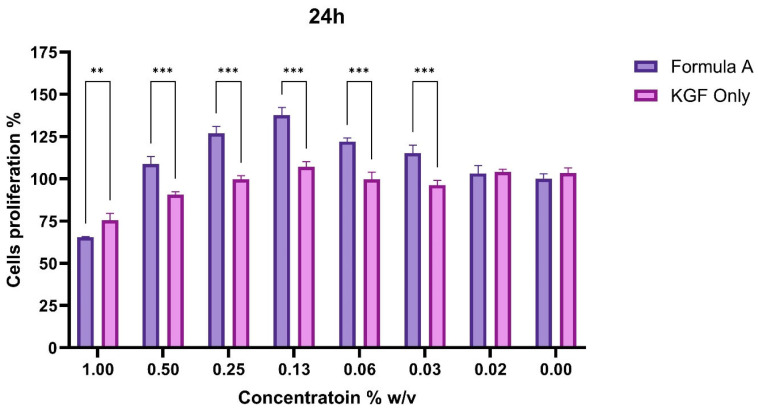
Percentage of fibroblast proliferation in response to mupirocin-KGF and KGF across concentration gradients at the 24-h time point. (** *p* < 0.01, *** *p* < 0.001).

**Figure 7:**
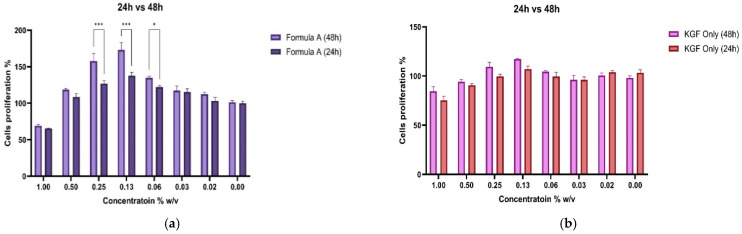
Comparative proliferation effects of mupirocin-KGF (**a**) and KGF (**b**) at 24 and 48 h. (* *p* < 0.05, *** *p* < 0.001).

To assess the functional impact of PAMAM dendrimer encapsulation on the bioactivity of Mu, fibroblast proliferation assays were conducted. The CCK-8 assay revealed that, compared with untreated control and KGF-treated cells, fibroblasts treated with the mupirocin-KGF formulation exhibited significantly greater viability at both 24 and 48 h. The increase in cell viability suggested that the PAMAM dendrimer improved drug solubility and bioavailability, ensuring sustained drug release over time. The proliferation data also confirmed that the mupirocin-KGF formulation did not induce cytotoxicity at therapeutic concentrations, supporting its safety for further wound healing applications. The increased fibroblast viability observed after 48 h (Figure 6 and Figure 7) suggests that the formulation not only supports cellular proliferation but also may provide additional benefits, such as increased nutrient uptake and cellular metabolic activity, both of which are crucial for tissue regeneration.

**Wound Healing Migration Analysis**

**Figure 8:**
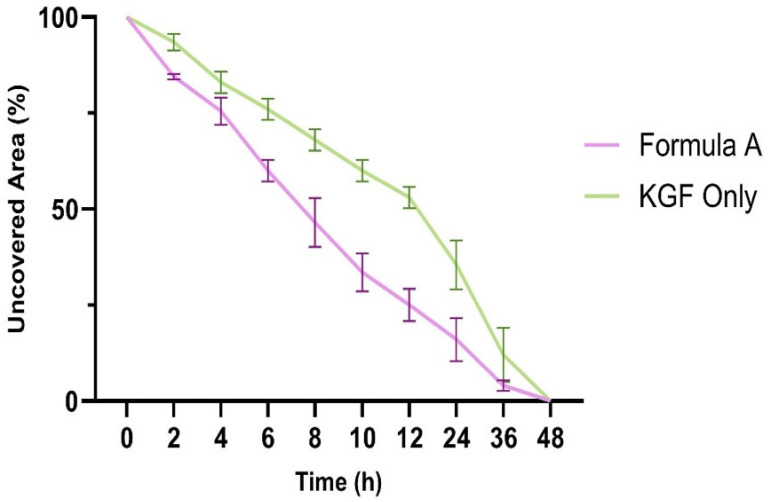
Scratch wound healing assay showing fibroblast migration over 48 h. Percentage of uncovered wound area plotted over time.

A scratch wound healing assay was conducted to evaluate fibroblast migration capacity, with measurements taken over 48 h (Figure 8). The wound closure patterns revealed distinct kinetics across the treatment groups, although all the conditions achieved complete closure within 48 h.

**Figure 9:**
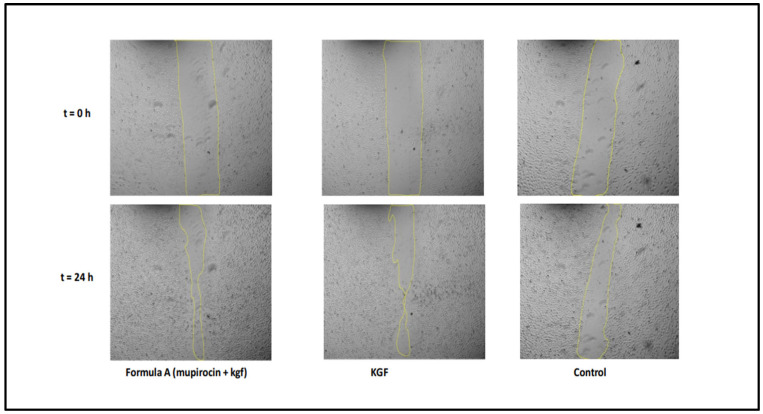
Representative phase-contrast images of individual fibroblast migration at t = 0 and 24 h posttreatment.

**Figure 10:**
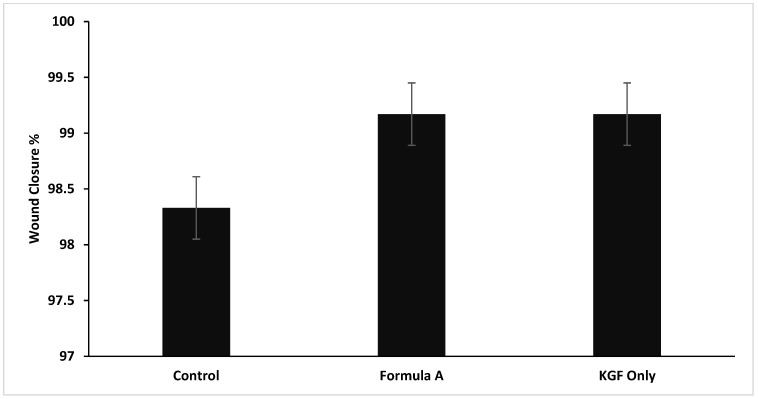
Wound closure as a percentage for the treatment groups compared with the control group from t = 0 to t = 24 h in the in vitro scratch assay.

The results demonstrated that the formulation significantly increased fibroblast migration rates compared with those of the untreated controls, indicating that the formulation has the potential to accelerate the wound healing process. The time to 50% wound closure was significantly shorter (8.0 ± 0.5 h) in the mupirocin-KGF-treated group than in the untreated control group (24 ± 1.8 h, *p* < 0.001), demonstrating a rapid response to the formulation. The average migration rate over 48 h was consistently greater in the mupirocin-KGF group (2.04 ± 0.15%/h) than in the control group (1.56 ± 0.12%/h, *p* < 0.001). This sustained enhancement indicates that the formulation supports prolonged cell migration rather than a short-lived burst effect. The formulation maintained comparable efficacy to KGF-treated fibroblasts, suggesting that it provides similar promigratory benefits while also delivering antimicrobial action. These findings underscore the dual role of mupirocin-KGF in preventing bacterial infections at the wound site and actively promoting fibroblast migration and tissue repair (Figure 9 and Figure 10). The ability of the formulation to enhance fibroblast migration is particularly significant, as rapid cell migration is a key step in wound closure and epithelialization [22].

The migration rate analysis revealed biphasic patterns across treatments, with higher rates during the initial phase followed by a gradual decrease. Formulation A significantly increased early migration rates, particularly during the 6–8 h interval, where it achieved maximal activity.

The overall migration parameters (Table 4) further confirmed the excellent wound healing capacity of the formulation. The time to 50% closure was significantly shorter in the formulation A treatment group than in the control group (*p* < 0.001), but the efficacy of formulation A was comparable to that of treatment with KGF alone. The average migration over 48 h was greater with formulation A (2.04 ± 0.15%/h) than with the control formulation (1.56 ± 0.12%/h, *p* < 0.001), demonstrating consistent improvement in the wound healing capacity throughout the observation period.

**Flow Cytometry Analysis**

To further understand the biological effects of the mupirocin-KGF formulation, flow cytometry analysis was performed to evaluate the expression of wound healing markers, including collagen I and α-smooth muscle actin (α-SMA).

**Figure 11:**
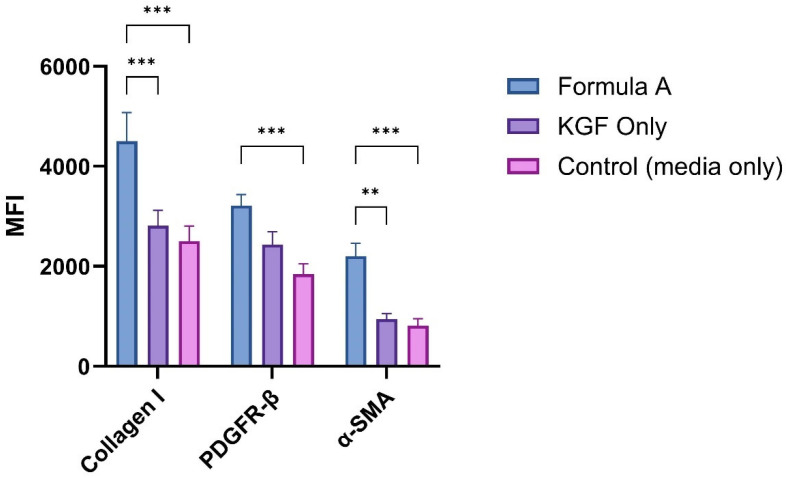
Expression of collagen 1, PDGFR-β and α-SMA in the formulation A- and KGF-treated groups compared with the control group. The values represent the mean fluorescence intensity (MFI) ± SD from three independent experiments (n = 3). Statistical significance is indicated by asterisks (** *p* < 0.01, *** *p* < 0.001) and was determined via two-way ANOVA followed by Tukey’s multiple comparisons test.

The expression of key fibroblast-associated proteins was evaluated in HS27 cells following a 48 h treatment with formulation A, KGF only or the control. Three critical markers, namely, collagen 1, an extracellular matrix protein; PDGFR-β, a cell surface receptor; and α-SMA, a myofibroblast differentiation marker, were investigated. PDGFR-β expression (Figure 11) was significantly greater in the formulation A group (3210 ± 229 MFI) than in the control group (1842 ± 211 MFI, *p* < 0.001). Compared with the control group, the KGF-treated group presented slightly greater expression (MFI of 2200 ± 262), with no significant difference. Collagen 1 expression was significantly greater in the formulation A-treated group (4500 ± 577 MFI) than in the control group (2500 ± 305.1 MFI, *p* < 0.001). Compared with the control group, the KGF-treated group also presented significantly greater collagen 1 expression (2811 ± 312.8 MFI, *p* < 0.001). Notably, the collagen I-inducing capacity of formulation A was comparable to that of the KGF-treated group, with no significant difference between these treatments (*p* = 0.19). α-SMA expression patterns were significantly greater in the formulation A-treated group (2200 ± 261 MFI) than in the control group (810 ± 140 MFI, *p* < 0.001). There was no significant difference between the KGF-only treatment group and the control group. In contrast, formulation A significantly differed from the KGF-treated group X (944 ± 114 MFI, *p* < 0.01).

The upregulation of collagen I suggests enhanced extracellular matrix deposition, a critical process for restoring the structural integrity of damaged tissue. Increased α-SMA expression indicates fibroblast differentiation into myofibroblasts, which play a key role in wound contraction and matrix remodelling. The observed changes in fibroblast marker expression suggest that the formulation not only enhances cell proliferation and migration but also triggers cellular responses that are essential for effective wound healing. Taken together, these results confirm that the PAMAM dendrimer serves as an efficient delivery platform that ensures both sustained Mu release and fibroblast activation to accelerate tissue regeneration.

**Kirby-Bauer disk diffusion test**

Mu is encapsulated in the hydrophobic cavity of HP-β-CD to create an HP-β-CD-Mu complex, which may improve Mu solubility and stability in aqueous solution. However, when KGF-3G PAMAM dendrimers are added to aqueous solution, HP-β-CD-Mu-KGF-PAMAM-NPs are created. These formed NPs were then transformed into HPMC nanogels.

In antimicrobial studies, HP-β-CD effectively increased Mu solubility in Mueller–Hinton agar medium, potentially leading to improved antibacterial efficacy against *S. aureus*, *E. coli*, and MRSA, as shown in Table 5. Compared with the positive control, the mupirocin-KGF formulation resulted in a greater inhibition zone against *S. aureus*, indicating enhanced efficacy. Additionally, while *E. coli* exhibited lower susceptibility to the positive control because its outer membrane acts as a barrier to antibacterial agents, the mupirocin-KGF formulation significantly improved antibacterial activity against this Gram-negative bacterium [23]. This improvement may be attributed to the presence of G3 PAMAM dendrimers, which enhance bacterial membrane permeation, thereby increasing Mu sensitivity.

Similarly, the mupirocin-KGF formulation exhibited notable antibacterial activity against MRSA, with inhibition zones comparable to those of the positive control. These results suggest that the formulation may be effective against antibiotic-resistant strains such as MRSA.

According to Azadeh Serri et al., vancomycin-PAMAM dendrimers have strong antibacterial activity against Gram-negative bacteria, causing vancomycin MICs in *E. coli* to decrease by approximately twofold [24]. This finding aligns with the enhanced efficacy observed in the mupirocin-KGF formulation. Given its broad antibacterial potential, this formulation may be a promising candidate for topical applications in treating skin infections caused by *S. aureus*, *E. coli*, and MRSA.

## 3. Materials and Methods

**Materials**

Mupirocin (Sigma, Livonia, MI, USA), PAMAM dendrimer (Sigma, Livonia, MI, USA), Keratinocyte Growth Factor human (Sigma, Livonia, MI, USA), Hydroxypropyl β-cyclodextrin (Sigma, Livonia, MI, USA), Disposable Sterile Petri Dish, (Brandon, Hillsborough County, FL, USA), Dulbecco’s modified Eagle medium (Thermo fisher, Waltham, MA, USA), L-glutamine (Thermo fisher, Waltham, MA, USA), trypsin-EDTA (Thermo fisher, Waltham, MA, USA), Accutase solution sterile-filtered (Sigma, Livonia, MI, USA), Anti-Collagen I antibody (Abcam, Cambridge, UK). All the reagents purchased were of either analytical grade or cell culture grade and were used as received.

**Molecular Docking Analysis**

The AutoDock Vina-Extended SAMSON software platform V4.0.3 was used to carry out molecular docking. After the enzyme binding sites were identified on the basis of interactions with their natural ligands, a cleaning and optimization procedure was carried out. SAMSON software was used to minimize the number of ligands (Et = 0.025 kcal/mol). Flexible docking was used to create ten positions for every docking exercise after water molecules were eliminated. Each ligand’s highest-ranking posture was chosen, and the ligand–protein interaction analyser extension was used to look for protein–ligand interactions [25,26].

**Preparation of the Mupirocin-KGF Formulation**

To formulate the mupirocin–KGF nanoparticle complex, 123.2 mg of hydroxypropyl β-cyclodextrin (HP-β-CD) and 200 mg of mupirocin (Mu) were accurately weighed and completely dissolved in 5 mL of 10% ethanol using a vortex mixer. This approach facilitated the formation of an inclusion complex (HP-β-CD-Mu), consistent with established cyclodextrin-based drug encapsulation techniques [27]. The solution was subjected to vacuum drying at 200 psi and 50 °C to remove solvent residues. Subsequently, 9.8 mL of distilled water was added to the dried residue, followed by sonication for 30 min to ensure full rehydration and dispersion.

A keratinocyte growth factor (KGF)–PAMAM dendrimer complex—comprising 2 mg of PAMAM, 15 ng of KGF, and 0.2 mL of glycerol—was then incorporated into the cyclodextrin-based mixture. The use of PAMAM dendrimers to stabilize and deliver therapeutic biomolecules is well-established in nanoparticle engineering [28]. The resulting hybrid nanoparticle system (HP-β-CD-Mu-KGF-PAMAM-Nps) integrates mucoadhesive, controlled-release, and synergistic therapeutic components. Finally, 500 mg of hydroxypropyl methylcellulose (HPMC) was added as a thickening and stabilizing agent, and the mixture was stirred magnetically for 30 min to complete the formulation of the mupirocin–KGF nanoparticle delivery system.

**Particle size and zeta potential measurements**

The zeta potential, hydrodynamic size and polydispersity index (PDI) of the mupirocin-KGF formulation were measured via Doppler anemometry via a zeta-sizer (Malvern instrument, Worcestershire, UK) and analysed via Malvern v7.02 software [29]. The mupirocin-KGF formulations were diluted with 0.9% sodium chloride solution until they contained less than 2% mupirocin-KGF formulation to ensure proper dispersion of the mupirocin-KGF formulation by adding 0.5 mL of nanogel with 9.9 mL of 0.9% sodium chloride solution before they were scanned under a Malvern Zetasizer nano ZS (Worcestershire, UK). Furthermore, the morphological characteristics of the HP-β-CD-Mu-KGF-PAMAM microparticles were determined via field emission scanning electron microscopy (FESEM), Brand/Model: Carl Zeiss (Oberkochen, Germany)/GeminiSEM 500.

**Determination of the surface tension of the mupirocin-KGF formulation**

To measure surface tension via a dual Nouy tensiometer, the instrument ring was cleaned, and a 100 mL beaker was filled with water. The tensiometer is zero, and the ring is positioned in the center to eliminate interference. The ring was lifted to break free of the liquid surface to capture the water surface tension. The process was repeated to determine the surface tension of the mupirocin-KGF formulation, swapping out the water for the nanogel.

**In Vitro membrane permeation studies using a Franz diffusion cell**

Membrane permeation of mupirocin release from the mupirocin-KGF formulation was performed in a Franz diffusion cell system with a 1.77 cm^2^ diffusion area and a 12 mL capacity of the receptor compartment. Franz cells were prepared by inserting the polymer membrane between the donor and receptor compartments. Then, 0.3 g of the 2% mupirocin in the mupirocin-KGF formulation (*w*/*w*) formulation was added to the donor compartment. The receiver compartment was filled with 12 mL of acetate buffer (pH 5.5). The receptor mixture was continuously stirred at 300 rpm while being kept at 32 ± 1 °C. At various time intervals of 5, 30, 60, 90, 120, 150, and 180 min, aliquots (1.0 mL) were extracted. To maintain the sink condition, the same quantity was returned to the receiver compartment after each aliquot was removed. The tests were carried out six times (*n* = 6), and the results are expressed as the mean values ± standard deviations (SDs) for the membranes. The amount of mupirocin in the collected medium was determined via high-performance liquid chromatography. Briefly, HPLC equipment (Waters 2487 Dual λ Absorbance Detector with Water 1515 Isocratic HPLC Pump and Supelco^®^ Analytical column with 15 cm × 4.6 mm i.d.; 5 µm particle size) will be used to measure the amount of mupirocin. Methanol and ultrapure water (90:10) were combined to provide the mobile phase for gradient elution, with a flow rate of 1 mL/min. Ten microlitres of the sample will be introduced into the chromatographic apparatus. The wavelength used for detection was 220 nm.

The cumulative amount of mupirocin per surface area (μg/cm^2^) was calculated and plotted as a function of time [30]. The percentage of released drug was calculated via the following equation [31,32]:

$$Drug\;release\left(\%\right)=\frac{Rt}{L}\;\times 100$$

where L is the initial quantity of mupirocin in the formulation and Rt is the total amount of mupirocin released at time t.

**Cell Culture and Treatment Conditions**

The American Type Culture Collection human dermal fibroblasts (HS27, Model: ATCC^®^ CRL-1634™) were cultured in high-glucose Dulbecco’s modified Eagle medium (DMEM, Gibco #11965092) supplemented with 10% heat-inactivated fetal bovine serum (FBS, Gibco #16140071), 1% penicillin/streptomycin (100 U/mL penicillin, 100 μg/mL streptomycin, Gibco #15140122), and 2 mM L-glutamine (Gibco #25030081). Cells were cultured at 37 °C with 5% CO_2_ and 70–80% confluence, then passaged using 0.25% trypsin-EDTA (Gibco #25200056). Experiments used cells from passages 4–8. Cells between passages 4 and 8 were used for all experiments. All gel formulations (KGF-Mu & Mu only) were prepared under sterile conditions. Stock solutions were prepared at 10% *w*/*v* in sterile PBS. Working dilutions were prepared fresh in complete culture medium immediately before use.

**Cell Proliferation Assay**

**Cell seeding and treatment**

HS27 cells were seeded in flat-bottom 96-well tissue culture plates (Corning #3595) at a density of 5 × 10^3^ cells/well in 100 µL of complete medium. After 24 h of attachment (approximately 30% confluence), the medium was aspirated and replaced with 100 µL of treatment dilutions. Serial dilutions were prepared in complete medium starting from 1% *w*/*v*, with 1:2 dilutions down to 0.016% (final concentrations: 1%, 0.5%, 0.25%, 0.125%, 0.0625%, 0.031%, 0.016%). The control wells received medium only [18,19]. Each condition was tested in triplicate columns.

**CCK-8 assay procedure**

At 24 and 48 h posttreatment, 10 µL of CCK-8 solution (Dojindo #CK04) was added to each well. The plates were incubated for 2 h at 37 °C in the dark. The absorbance was measured at 450 nm with reference to 650 nm via a Tecan Infinite M200 PRO microplate reader. Background values (CCK-8 in medium without cells) were subtracted from all readings.

**Scratch Wound Healing Assay**

**Cell Preparation**

HS27 cells were seeded in 24-well plates (Corning #3513) at 1 × 10^6^ cells/well in 1 mL of complete medium. After reaching 90% confluence, the cells were serum-starved (0.5% FBS) for 12 h to minimize the effects on proliferation.

**Wound creation**

Using a sterile 200 µL pipette tip held at a 45° angle, a straight scratch was made across the cell monolayer. A guideline was drawn on the bottom of the plate to ensure consistent image acquisition. The wells were washed twice with 1 mL of PBS (37 °C) to remove detached cells. Treatment media containing optimal concentrations of test formulations (determined from the proliferation assay) were added to low-serum medium (0.5% FBS).

**Image acquisition**

Images were captured every 2 h for 48 h via an Olympus IX73 inverted phase-contrast microscope equipped with a DP74 digital camera (10× objective, 0.30 NA). The temperature and CO_2_ were maintained in a stage-top incubator (Tokai Hit). Three random fields per well were photographed at consistent positions relative to the guideline. ImageJ software (version 1.53k, National Institutes of Health, Bethesda, MD, USA) was used for wound area measurements. Images were processed using the following protocol where firstly the images were converted to 8-bit grayscale. Then the wound area was outlined manually using the polygon selection tool. The area of measurements was recorded in square micrometers. It is followed by three random fields per well were analyzed and averaged.

Calculation of Migration Rate

The formula as follows:

$$\mathrm{Wound}\;\mathrm{Closure}\;\mathrm{Percentage}\;(\%)\;=\;\frac{A0-At}{A0}\;\times \;100$$

The following formula was used to calculate the average migration rate:

$$\mathrm{Migration}\;\mathrm{Rate}\;(\mu m/h)\;=\;\frac{Initial\;wound\;width-Final\;wound\;width}{2\times Time\;in\;hours}$$

The division by 2 accounts for cell migration from both edges of the wound.

While the following formula was used to calculate wound closure percentage:

Wound Closure (%) = [(Area at 0 h—Area at time t)/Area at 0 h] × 100

where Area at 0 h is the initial wound area immediately after scratch creation, and Area at time t is the wound area at the measured time point.

**Flow Cytometry Analysis**

**Sample Preparation**

After 48 h of treatment, the cells were harvested with Accutase solution (Sigma #A6964, 10 min at 37 °C). The cells were counted, and 1 × 10^6^ cells per condition were aliquoted into FACS tubes. The cells were washed twice with cold PBS + 1% BSA (flow buffer).

**Fixation and Permeabilization**

The cells were fixed with 4% paraformaldehyde (freshly prepared) for 15 min at room temperature. After being washed with flow buffer, the cells were permeabilized with 0.1% Triton X-100 in PBS for 10 min at room temperature. Blocking was performed with 5% BSA for 30 min.

**Antibody Staining**

For immunostaining analysis, the cells were incubated with a panel of primary antibodies, including antibody against Collagen Type 1 (Abcam ab34710, 1:100 dilution), antibody against platelet-derived growth factor receptor beta (Cell Signalling #3169, 1:100 dilution), and antibody against alpha-smooth muscle actin (Sigma A5228, 1:100 dilution) antibodies. The primary antibody mixture was incubated for 1 h at 4 °C in flow buffer. Both Alexa Fluor 488-conjugated goat anti-rabbit (Invitrogen #A11008, 1:500 dilution) and Alexa Fluor 647-conjugated goat anti-mouse (Invitrogen #A21235, 1:500 dilution) antibodies were used to incubate the samples for 30 min at 4 °C in the dark after they were thoroughly washed.

Flow cytometric analysis was performed on a BD FACSCanto II cytometer configured with specific voltage settings (FSC: 250, SSC: 400, FITC: 500, APC: 600). Proper compensation was established via single-stained controls, and a minimum of 10,000 events were recorded per sample. Data analysis was executed via FlowJo software (version 10.8), following a systematic gating strategy that included the initial selection of live cells on the basis of FSC/SSC parameters, exclusion of doublets through FSC-H/FSC-A analysis, establishment of positive population gates via fluorescence minus one (FMO) controls, and subsequent calculation of the mean fluorescence intensity (MFI) and percentage of positive cells.

**Kirby-Bauer disk diffusion test**

The materials used for the Kirby–Bauer disk diffusion test included a bacterial strain Staphylococcus aureus ATCC 43300 MRSA (Microbiologics, USA). obtained from the Department of Medical Microbiology, Faculty of Medicine and Health Sciences, University Putra Malaysia (UPM) and identified via standard microbiological methods. Mueller–Hinton agar (MHA), a nonselective, nutrient-rich medium suitable for antimicrobial susceptibility testing (HiMedia, India), was used. Commercially available antibiotic-impregnated disks (Oxoid, UK) with standardized concentrations were employed for testing. Sterile cotton swabs were utilized for uniform inoculation of the bacterial culture, whereas sterile forceps were used for handling the antibiotic disks aseptically. An inoculating loop was used for bacterial culture transfer, and sterile saline (0.85% NaCl) was used to adjust the bacterial suspension to the 0.5 McFarland standard. The McFarland standard (0.5 turbidity) served as a reference standard for bacterial suspension density (BD, USA). The incubator was set at 37 °C for 16–18 h to allow bacterial incubation, and a calliper or ruler was used to measure the zone of inhibition accurately. The Kirby–Bauer disk diffusion method was performed according to the Clinical and Laboratory Standards Institute (CLSI) guidelines (CLSI, 2020). MHA was prepared according to the manufacturer’s instructions and autoclaved at 120 °C for 15 min. The medium was poured into sterile Petri dishes (90 mm diameter) to a depth of approximately 4 mm and allowed to solidify under aseptic conditions. Five milliliters of sterile saline were used to emulsify a new bacterial colony that was 18–24 h old. The turbidity of the suspension was adjusted to meet the 0.5 McFarland standard, which is equivalent to approximately 1.5 × 10^8^ CFU/mL. After a sterile cotton swab was dipped into the bacterial mixture, the swab was pressed against the inner wall of the tube to remove any extra liquid. To guarantee a consistent bacterial lawn, the swab was then used to streak the whole MHA plate surface in three different orientations. Before the antibiotic disks were applied, the plate was allowed to dry for five minutes. Empty disks were placed on the agar surface via sterile forceps, ensuring even spacing (at least 24 mm apart) to prevent overlapping of inhibition zones. After loading 10 µL of sample onto each disk, the plates were incubated inverted at 37 °C for 16–18 h. After incubation, the diameter of each inhibition zone was measured in millimetres via a ruler. The results were interpreted according to CLSI guidelines (CLSI, 2020) by comparing zone diameters with standard breakpoints for resistance and susceptibility [33].

**Quality Control**

To ensure accuracy, control strains such as *Escherichia coli* ATCC 25922 and *Staphylococcus aureus* ATCC 25923 were used. The temperature and incubation time were strictly maintained to ensure reproducibility, and each test was performed in triplicate for consistency [34].

**Statistical analysis**

All the experiments were performed in technical triplicate and were repeated in three independent biological replicates. The data are presented as the means ± standard deviations (SDs). Statistical analysis was performed via GraphPad Prism 10.0 software. A normal distribution was verified via the Shapiro–Wilk test. One-way ANOVA followed by Tukey’s post hoc test was used for multiple comparisons. Two-way ANOVA with Bonferroni correction was applied for time-course analyses. All *p*-values reported are adjusted for multiple comparisons using the respective post hoc corrections. The *p* values < 0.05 were considered statistically significant. Effect sizes were calculated via Cohen’s d.

## 4. Conclusions

This study establishes a mupirocin-KGF hydrogel formulation as a promising dual-functional therapeutic agent that combines antimicrobial activity with enhanced wound healing efficacy. Molecular docking analyses confirmed the stable interaction of the mupirocin-KGF complex with isoleucyl-tRNA synthetase, indicating its potential to maintain and improve the antibacterial function of mupirocin. The in vitro assessments further demonstrated significant improvements in fibroblast proliferation, migration, and extracellular matrix remodelling, all of which are crucial for effective wound healing. The sustained drug release profile, coupled with favourable physicochemical properties, underscores the stability and controlled bioavailability of the formulation. Additionally, antimicrobial evaluations confirmed its potent efficacy against both Gram-positive and Gram-negative bacteria, including MRSA, supporting its potential application in infected wound management. The synergistic interaction between mupirocin and KGF highlights the advantage of integrating antimicrobial and regenerative properties in a single formulation, addressing both infection control and tissue repair. Overall, these findings suggest that the mupirocin-KGF formulation could significantly advance wound healing therapies, particularly for infections involving antibiotic-resistant pathogens. Further in vivo studies are warranted to validate these results and optimize formulation parameters for clinical translation.

The lack of in vivo validation and the possible variability of KGF in biological settings, which could impact its therapeutic efficacy, among the study’s limitations. In vivo research to promote clinical translation in advanced wound care applications and formulation optimization for improved growth factor stability will be the main goals of future work.

## Figures and Tables

**Table 1:** Molecular docking results showing the binding affinities and key interacting residues for PAMAM-Mu, Mu alone, and the PAMAM dendrimer complexes.

Receptor	Ligand	Affinity (kcal/mol)	H-Bonding/Hydrophobic Interactions	
			Residues	Distance (A)
Isoleucyl-tRNA synthetase	Mupirocin-KGF complex	−7.66223	ASP 612/O (O)	3.19537
			TYR 398/O (O)	3.23397
			GLN 828/O (O)	2.80594
			PHE 726/O (O)	3.37672
			ARG 826/O (O)	3.26878
Isoleucyl-tRNA synthetase	Mupirocin	−7.58297	LYS 648/NZ (N)	3.22193
	PAMAM dendrimer + Mu	−6.41783	C1266H2536N506O252 1/N (N)	2.95777
			C1266H2536N506O252 1/N (N)	3.30448
			C1266H2536N506O252 1/N (N)	2.94172
			C1266H2536N506O252 1/C (C)	3.42974
			C1266H2536N506O252 1/O (O)	3.21500
			C1266H2536N506O252 1/O (O)	3.36233
			C1266H2536N506O252 1/O (O)	3.41635

**Table 2:** Zeta analysis of the mupirocin-KGF formulation. The data are presented as the means ± SDs of three independent experiments where significant differences in size, PDI and zeta potential were all observed between all groups, with *p* < 0.05 via one−way ANOVA with a post hoc test (Tukey) via SPSS 11.

Samples	Size, nm	PDI	Zeta Potential (mV)
Mupirocin-KGF formulation	119.2 ± 3.43	0.286 ± 0.035	−24.79 ± 0.35

**Table 3:** Surface tension measurements of the mupirocin-KGF formulation compared with the control, highlighting its wetting properties.

Sample	Surface Tension (dyne/cm)			
	1st Reading	2nd Reading	3rd Reading	Mean ± Standard Deviation
MuGel Gel	24.8	24.6	24.7	24.7 ± 0.1

**Table 4:** Comparative analysis of migration kinetics, including time to 50% closure, total migration rates, and final closure time.

Parameter	Control	Formulation A	KGF Only
Total Closure (%)	100	100	100
Time to 50% (h)	24 ± 1.8	8.0 ± 0.5	6 ± 0.4
Average Rate (%/h)	1.56 ± 0.12	2.04 ± 0.15	2.08 ± 0.16
Initial Rate (0–12 h)	2.5 ± 0.21	6.5 ± 0.48	6.83 ± 0.52
Final Rate (36–48 h)	0.83 ± 0.05	0.25 ± 0.02	0.17 ± 0.01

**Table 5:** Antibacterial efficacy of mupirocin-KGF against *S. aureus*, *E. coli*, and MRSA on the basis of zone of inhibition measurements.

Samples	Bacterial Strain	Amount (µL)	Diameter of Zone of Inhibition (mm)
Mupirocin-KGF formulation	*E. coli*	10 (30 µg mupirocin)	24
	MRSA	10 (80 µg mupirocin)	30
	*S. aureus*	10 (30 µg mupirocin)	34
Negative Control	*E. coli*	10 (30 µg mupirocin)	0
	MRSA	10 (80 µg mupirocin)	0
	*S. aureus*	10 (30 µg mupirocin)	0
Positive Control	*E. coli*	10 (30 µg mupirocin)	16
	MRSA	10 (80 µg mupirocin)	32
	*S. aureus*	10 (30 µg mupirocin)	31

## Data Availability

Data are contained within the article and Supplementary Materials.

## Supplementary Materials

The following supporting information can be downloaded at: https://www.mdpi.com/article/10.3390/molecules30234523/s1, Figure S1: Zeta potential distribution of the HP-β-Cy-Mu-FGF-PAMAM-Nps particle in the Mu-FGF nanogel; Figure S2: HP-β-Cy-Mu-FGF-PAMAM-Nps particle size distribution in the Mu-FGF nanogel.
