# Supplementary material for: Biological Characterization of Mupirocin–KGF Hydrogel and Its Regenerative Potential in Human Fibroblast-Mediated Wound Healing
Source: Molecules. 2025 Nov 23;30(23):4523. doi: 10.3390/molecules30234523 (PMC12693590; doi:10.3390/molecules30234523)
Supplement: Supplementary file 1 [file molecules-30-04523-s001.zip › molecules-3960309-supplementary.pdf]

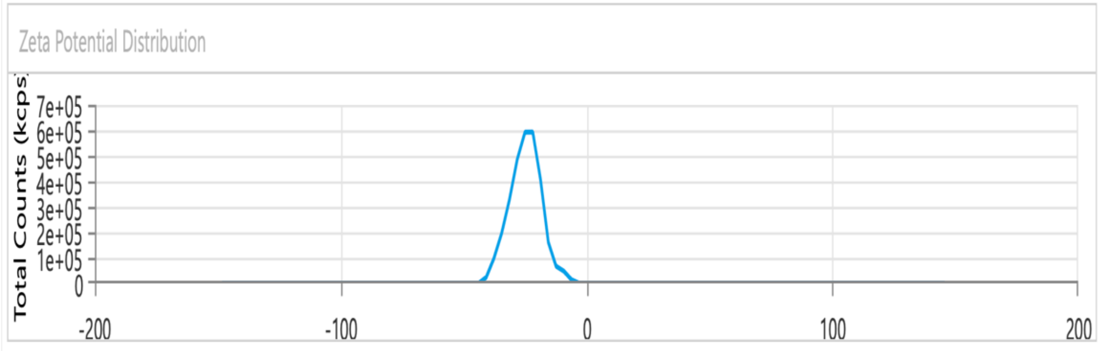

Figure: Zeta potential distribution of the HP-β-Cy-Mu-FGF-PAMAM-Nps particle in *the* Mu-FGF nanogel

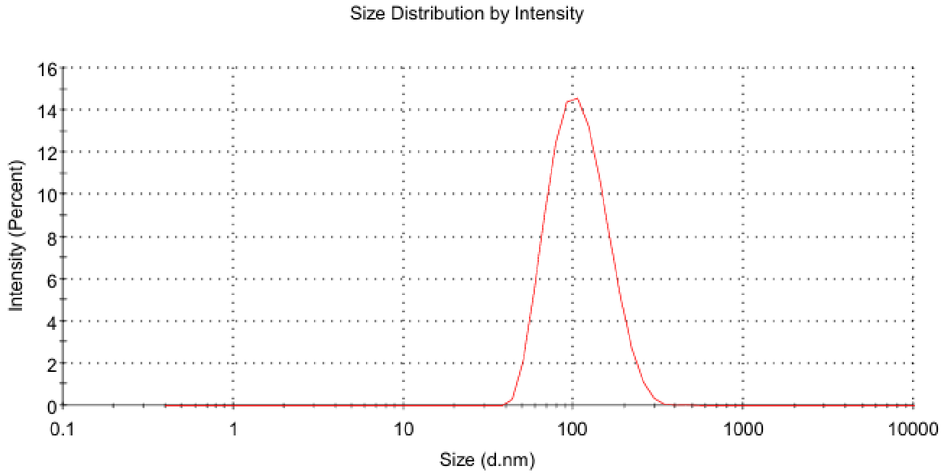

Figure*:* HP-β-Cy-Mu-FGF-PAMAM-Nps particle size distribution in the Mu-FGF nanogel
